# Mixed Transportation Network Design under a Sustainable Development Perspective

**DOI:** 10.1155/2013/549735

**Published:** 2013-02-17

**Authors:** Jin Qin, Ling-lin Ni, Feng Shi

**Affiliations:** ^1^School of Traffic and Transportation Engineering, Central South University, Changsha 410075, China; ^2^Dongfang College, Zhejiang University of Finance and Economics, Hangzhou 310012, China

## Abstract

A mixed transportation network design problem considering sustainable development was studied in this paper. Based on the discretization of continuous link-grade decision variables, a bilevel programming model was proposed to describe the problem, in which sustainability factors, including vehicle exhaust emissions, land-use scale, link load, and financial budget, are considered. The objective of the model is to minimize the total amount of resources exploited under the premise of meeting all the construction goals. A heuristic algorithm, which combined the simulated annealing and path-based gradient projection algorithm, was developed to solve the model. The numerical example shows that the transportation network optimized with the method above not only significantly alleviates the congestion on the link, but also reduces vehicle exhaust emissions within the network by up to 41.56%.

## 1. Introduction

Sustainable development, which is generally defined as “development that meets the needs of the present without compromising the ability of future generations to meet their own needs” [1], has in recent years become a widely appreciated concept with strong political and popular support. There are many components to this notion, such as economy, society, resources, and the environment. In particular, followed with the improvement of urbanization, the conflicts between transportation and the environment are becoming more and more serious. Therefore sustainable transport, which is defined as “satisfying current transport and mobility needs without compromising the ability of future generations to meet these needs” [2], has in recent decades become a very important goal in the field of transportation engineering. 

The transportation network is one of the most critical infrastructures in urban structures and plays an essential role in meeting normal city operation. It is regularly improved to cope with the ever-growing demands in travel and problems of congestion. In order to optimize some given performance measures (e.g., total travel time or generalized cost) of the network, a crucial decision in the improvement of the network is the allocation of the limited resources to the capacity expansion of the existing links and/or the addition of new candidate links. Such decision problem is always referred to as a transportation network design problem (TNDP). 

Morlok firstly proposed the quantitative TNDP in 1973, and since then it has been widely studied by researchers over the last 40 years. The TNDP is always formulated as a mathematical programming model with traffic equilibrium assignment, which can be categorized into three classes: continuous transportation network design problem (CTNDP), discrete transportation network design problem (DTNDP), and mixed transportation network design problem (MTNDP). CTNDP determines the optimal continuous expansion of capacity to existing links [3–8]. DTNDP selects the optimal link additions from a set of candidate links [9–12]. MNDP is a mixture of CTNDP and DTNDP, which simultaneously incorporates both discrete and continuous decision variables [13, 14]. Obviously, MTNDP is more sensible than others in the description of the practical transportation design problem. Nevertheless, MTNDP deals with discrete variables and continuous variables at the same time, so it is very difficult to solve. 

Although there is much literature on transportation network design, we can easily find that their common objective is to minimize related economic costs, and goals related to sustainable development are always not taken into account. Guided by this transportation network design theory, the development of transportation engineering has brought about serious consequences regarding the ecological environment in recent decades. Given this situation it is very important to develop a study on the methods and theories of sustainable transportation network design.

 This paper mainly studies MTNDP in the consideration of sustainable development, in which the sustainable factors, such as car exhaust emissions or land used and link load are involved. The problem is described as a bilevel programming model and its solution method is proposed. 

The remainder of this paper is organized as follows. Hypothesis and notations are given in Section 2.  Section 3 formulates the bilevel programming model for the problem.  Section 4 designs a solution method based on the annealing algorithm and path-based gradient projection method. In Section 5, numerical experiments are conducted to test the proposed approach. The final section concludes the paper and briefly discusses future research directions.

## 2. Notations

The complexity of the MTNDP lies mainly within the setting of link-state decision variables, because a single variable cannot describe the continuous link capacity and discrete link connection state at the same time. 

Nonetheless, we know that in reality it is meaningless to subtly improve the link capacity. The improvement of a link always comprises of adding new lanes or significantly increasing its capacity; namely, in practice the link capacity expansion is not continuous but within a limited status. Thus the link status can be divided into different discrete grade variables according to the link capacity, which could discretize the mixed problem and lay a foundation for the mathematical model formulation. 

For example, according to the Road Commission of China Association for Engineering Construction Standardization [15] the link states are usually divided into 5 grades: high-speed link, 1st-level link, 2nd-level link, 3rd-level link, and 4th-level link. Each link grade represents different lanes, different unit construction costs, different design speeds, and different capacities. On this occasion it is possible to discretize the mixed problem by using the link grade decision variables. 

It can be seen from Table 1 that we can set the link grade decision variable *u* with 10 integers, from 0–9 (in which 0 indicates that the link is not built) respectively representing the different link grades, meanwhile corresponding to the different link capacities and number of lanes, as shown in Table 1. So the link states in the mixed transportation network can be uniformly described by this link grade decision variable. The numbers of the decision variables need not to be restricted to the range of 0 to 9, and in practice can be set properly according to the needs.

In formulating the optimization model, the following notation is used:

**A**_1_: set of existing links in the network,
**A**_2_: set of candidate links to be added,
**A**_3_: set of links that are determined to be added,
**A**: set of all links in the network, **A** = **A**_1_ ∪ **A**_3_,
**R**: set of original nodes,
**S**: set of destination nodes,
*r*: origin node index, *r* ∈ **R**,
*s*: destination node index, *s* ∈ **S**,
*rs*: origin-destination (OD) pair index,
*a*: link index, *a* ∈ *A*,
*K*_*rs*_: set of the paths connecting OD pair *rs*,
*u*_*a*_: state variable of link *a* ∈ **A**; we assume that there are *n* + 1 grade states from 0 to *n* in the network, so *u*_*a*_ ∈ {0,1, 2,…, *n*}, in which *u*_*a*_ = 0 means link *a* will not be built,
*u*_*a*_^0^: the initial grade of link *a* ∈ **A**, *u*_*a*_^0^ ∈ {0,1, 2,…, *n*},
*l*_*a*_: length of link *a* ∈ **A** (in kilometers),
*q*_*rs*_: travel demand between OD pair *rs*,
*x*_*a*_: total traffic flow on link *a* ∈ **A**,
*c*_*a*_: capacity of link *a* ∈ **A** is determined by the link grade *u*_*a*_,
*v*_*a*_: design speed of link *a* ∈ **A** (kilometers/hour) is determined by the link grade *u*_*a*_,
*t*_*a*_^0^: free-flow travel time on the link *a* ∈ **A** (in minutes). And if *u*_*a*_^  ^ = 0,  *t*_*a*_^0^ = +*∞*,
*t*_*a*_: travel time function of link *a* ∈ **A** (in minutes) we use the BPR function as *t*_*a*_ = *t*_*a*_^0^ · (1 + *α*(*x*_*a*_/*c*_*a*_)^*β*^) (*α*, *β* are all given positive parameters),
*R*_max⁡_: maximum values of allowed link load degree for all links,
*p*_*a*_(*x*_*a*_): CO emission function of vehicles on the link *a* ∈ **A**,
*I*_*a*_(*u*_*a*_): construction cost function per unit length of link *a* ∈ **A** when its link grade is *u*_*a*_,
*E*: upper limit value of land amount which can be used
*e*(*u*_*a*_, *u*_*a*_^0^): land-use scale function of the link *a* ∈ **A**, namely the land-use scale of the link *a* is a function on the initial state *u*_*a*_^0^ and final state *u*_*a*_,
*I*_*a*_(*u*_*a*_, *u*_*a*_^0^): the construction cost function per unit length of the link *a* ∈ **A**, that is to say, the construction cost of link *a* is a function of its initial state *u*_*a*_^0^ and final state *u*_*a*_,
*f*_*k*_^*rs*^: traffic flow on path *k* connecting OD pair *rs*,
*δ*_*a*,*k*_^*rs*^: path/link incidence variables, if path *k* connecting OD pair *rs* passes link *a* ∈ **A**, then *δ*_*a*,*k*_^*rs*^ = 1, otherwise *δ*_*a*,*k*_^*rs*^ = 0.

## 3. Model Formulation

The TNDP consists in seeking a transportation network configuration that minimizes some objectives, subject to the equilibrium constraint.

### 3.1. Sustainable Development in Transportation Network Design

From the perspective of sustainable transport, we should take the sustainability factors into consideration in the design of the transportation network. Moreover, according to the definition of sustainable transport we could conclude that the sustainability factors in the transportation network should mainly focus on the pollution caused by vehicle exhaust emissions, resources utilization, and link load.

#### 3.1.1. Vehicle Exhaust Emissions

Vehicles continuously generate hazardous exhaust gases when they are running, which is the major cause of air pollution. In order to protect the ecological environment and human health, reducing vehicle exhaust emissions has been an ongoing endeavor of many governmental authorities over the past few decades.

Vehicle exhaust emissions contain a range of pollutants, such as carbon monoxide (CO), nitric oxide (NO), and nitrogen dioxide (NO_2_). Because it is almost solely emitted by vehicles, CO is considered as the indicator for the level of atmospheric pollution generated by vehicular traffic in the transportation network [16]. Thus if we can reduce one pollutant from vehicle emissions, other pollutants in the emissions will also be reduced. In this regard we only calculate CO as a control subject in this paper.

Obviously, vehicle exhaust emissions on the link depend on the link traffic flow. Yin and Lawphongpanich [17] proposed the following function to estimate vehicular CO emissions:

(1) $$\begin{array}{c} p_{a}\left(x_{a}\right)=0.2038\cdot t_{a}\cdot e^{0.7962(l_{a}/t_{a})}, \end{array}$$

where *l*_*a*_ is the length of link *a* ∈ **A** (in kilometers) and *t*_*a*_(*x*_*a*_) is the travel time for link *a* ∈ **A** (in minutes), and *p*_*a*_(*x*_*a*_) is the vehicular CO emissions on link *a* (in g/hour).

So the total CO emissions in the network are:

(2) $$\begin{array}{c} \sum_{a\in A}p_{a}\left(x_{a}\right)x_{a}=0.2038\times \sum_{a\in A}t_{a}\cdot x_{a}\cdot e^{0.7962(l_{a}/t_{a})}. \end{array}$$

From (2) it can be found that the total CO emission is associated with the vehicle travel time and the average travel speed on the link, so the CO emission in the network is difficult to determine. We could regard it as an environmental cost of the transportation network system, which should be minimized in the optimization objectives of the TNDP.

#### 3.1.2. Link Load

The service level of the transportation network perceived by travelers is generally the average travel speed or travel time. Obviously, the higher the average speed of the vehicles, the shorter the travel time, and the greater the perceived level of service. 

It is easy to know that the average speed, ${\overset{-}{v}}_{a}$, on the link, *a*, should be

(3) $$\begin{array}{c} {\overset{-}{v}}_{a}=\frac{l_{a}}{t_{a}}=\frac{l_{a}}{t_{a}^{0}\cdot \left(1+\alpha {\left(x_{a}/c_{a}\right)}^{\beta }\right)}=\frac{l_{a}}{t_{a}^{0}}\cdot \frac{1}{1+\alpha {\left(x_{a}/c_{a}\right)}^{\beta }}. \end{array}$$

Because *l*_*a*_, *t*_*a*_^0^, *α*, and *β* are all the parameters given, the average travel speed on the link, *a*, only depends on the value of *x*_*a*_/*c*_*a*_, which is usually known as the link load or congestion degree. Thus, to reach a certain service level, as well as to reserve some link capacity for future development, it is necessary to impose restrictions on the maximum link load.

According to the definition, *R*_max⁡_ is the maximum load allowed for all links in the transportation network, then the link load constraint can be expressed as

(4) $$\begin{array}{c} \frac{x_{a}}{c_{a}}\leq R_{\max}. \end{array}$$

#### 3.1.3. Land Resource Utilization

It is well known that urban land resources are limited and precious, which leads to the fact that they cannot be extravagantly used for traffic. A certain amount of land resource needs to be taken up when upgrading existing links or adding new links. To ensure sustainable development we should use as little land as possible on the premise that the expected goal is achieved. Therefore we should set an upper limit value for the amount of land used in the network design.

Obviously the amount of used land for the improvement of a link is relevant to the initial and final states of the link. According to the definition, the land-use scale function of link *a* is *e*(*u*_*a*_, *u*_*a*_^0^), thus the land-use scale constraint can be expressed as

(5) $$\begin{array}{c} \sum_{a\in A}e\left(u_{a},u_{a}^{0}\right)\leq E, \end{array}$$

where *E* is a given upper limit for the amount of land available in the network design.

#### 3.1.4. Construction Funds Utilization

That construction fund is also a limited resource. Therefore, with the premise of achieving the expected construction goal, the total construction cost should be as low as possible. Thus, the total construction cost ∑_*a*∈*A*_ *l*_*a*_ · *I*(*u*_*a*_, *u*_*a*_^0^) should be minimized in the problem, which is also to be minimized in the model.

### 3.2. Model Formulation

Similar to a conventional TNDP, the mixed sustainable TNDP can be formulated as a bilevel programming model, in which the upper-level model is the network structure design model and the lower-level model is the user equilibrium assignment model.

Mathematically, the formulation of the following bi-level programming model is

(6) min⁡ C=∑a∈A[θ·pa(xa)·xa+la·Ia(ua,ua0)],s.t.     Rmin⁡≤xaca≤Rmax⁡,  ∑a∈Ae(ua,ua0)≤E,   ua≥ua0,   ua∈{0,1,2,…,n},

where *x*_*a*_ is implicitly defined by the lower-level model:

(7) min⁡ T=∑a∈A∫0xata(xa,ca(ua))dx,s.t.  ∑k∈Krsfkrs=qrs, ∀s∈S,  ∀r∈R,  xa=∑r∈R∑s∈S∑k∈Krsfkrsδa,krs, ∀a∈A,  fkrs≥0, ∀s∈S,  ∀r∈R,  ∀k∈Krs.

In the upper-level model, the optimization objective is to minimize the total investment costs and vehicle emissions of CO at the same time, where parameter *θ* represents the unit conversion factor. Since the existing links generally will not be closed or degraded in the network improvement, it is possible to set the link grade restraint as

(8) $$\begin{array}{c} u_{a}\geq u_{a}^{0}. \end{array}$$

## 4. Method of Solution 

### 4.1. Simulated Annealing Algorithm

As mentioned previously, the MTNDP is NP-hard, and this nature makes heuristics the natural choice for it. In this paper we adopted the simulated annealing (SA) algorithm as the basic method to solve the model. 

SA is a probabilistic heuristic for global optimization problems for finding a good approximation to the global optimum of a given objective function in the search space. The strength of SA is illustrated by the breadth of studies found in areas such as logistics network design, computer network design, and machine scheduling, in which SA has been proven as an effective tool for approximating globally optimal solutions to many NP-hard problems.

Although SA studies abound, the area of transportation network design has not been addressed in the literature. It is from this point that the current study embarks.

**Figure 1:**
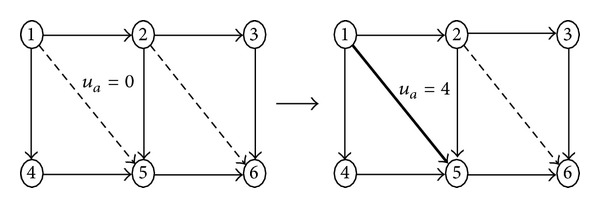
Neighboring function based on one link level being transformed.

**Figure 2:**
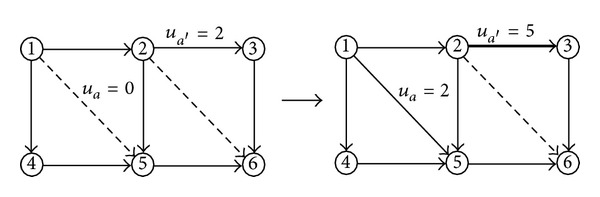
Neighboring function based on two link levels being transformed.

The neighborhood function plays a crucial role in the performance of SA. In order to improve the efficiency of local search we adopt neighborhood operations by simultaneously adjusting the grades of 1 or 2 links randomly in the neighborhood function. Figures 1 and 2 describe these two neighborhood operations respectively, which randomly select 1 or 2 links from the current network to conduct a link grade transformation operation (the solid lines represent the existing links, while the dashed lines represent the candidate links to be added). Note that the link grades can only be transformed into higher states.

In order to describe the procedure of the SA, $S,S^{\prime },\overset{-}{S}$ are used to represent the different solutions to the model, *C*(**S**) is used to represent the objective function value of the solution **S** in the upper-level model. Then the detailed steps of the SA algorithm can be described as follows.

**Step 1 (initialization)**

Set the initial and final temperature values as *T*_0_ and *t*_*f*_, respectively. The cooling rate *α*(0 < *α* < 1) is specified along with the maximum number of iterations *N* at each temperature value. Take the current network configuration as the initial solution **S** and the global optimal solution $\overset{-}{S}$. Define link set $\overset{-}{A}=\phi$. The iteration counter *i* = 0.

**Step 2 (equilibrium assignment)**

Conduct equilibrium assignment in the initial network and obtain the equilibrium link flows. Check all link load constraints in the upper-level model. If link *a* violates the constraint then $\overset{-}{A}=\overset{-}{A}\cup a$, and the penalty cost in the objective function should be imposed, that is $C\left(S\right)=C\left(S\right)+n\sum_{a\in \overset{-}{A}}l_{a}I\left(u_{a},0\right)$ (where *n* is the given positive integer). Set the global optimal solution $\overset{-}{S}=S$ and $C\left(\overset{-}{S}\right)=C\left(S\right)$.

**Step 3 (generate a feasible neighboring solution)**

Generate a neighboring solution **S**′ by altering the current solution **S** according to the given neighborhood function. Then check the feasibility of solution **S**′. If **S**′ is infeasible, it is necessary to repeat Step 3 to regenerate the neighboring solution; otherwise calculate *C*(**S**′) and proceed to Step 4.

**Step 4 (equilibrium assignment in a new network configuration)**

Conduct equilibrium assignment in the new network configuration (neighboring solution **S**′). Get the new equilibrium link flows. Reset $\overset{-}{A}=\phi$ and check the link load constraints in the upper model. If link *a* violates the link load constraints, then $\overset{-}{A}=\overset{-}{A}\cup a$ and $C\left(S^{\prime }\right)=C\left(S^{\prime }\right)+n\sum_{a\in \overset{-}{A}}l_{a}I\left(u_{a},0\right)$.

**Step 5 (evaluate the current solution with the neighboring solution)**

If $C\left(S^{\prime }\right)\leq C\left(\overset{-}{S}\right)$, $\overset{-}{S}=S$ as well as $C\left(\overset{-}{S}\right)=C\left(S\right)$. If *C*(**S**′) ≤ *C*(**S**), then set **S** = **S**′, *C*(**S**) = *C*(**S**′), proceed to Step 7, otherwise proceed to Step 6.

**Step 6 (examine the metropolis condition)**

The probability at which the relatively inferior neighboring solution should be accepted is *P*(**S**′) = exp⁡(−(*C*(**S**′) − *C*(**S**))/*T*_*i*_), where *T*_*i*_ is the current temperature. A random number *ρ* is then generated from the interval (0,1). If *ρ* < *P*(**S**′), then **S** = **S**′ and *C*(**S**) = *C*(**S**′).

**Step 7 (increment counters)**

*i* = *i* + 1. If *i* ≤ *N*, return to Step 3, otherwise proceed to Step 8.

**Step 8 (adjust the temperature)**

Adjust the temperature value by the cooling rate. Mathematically this is *T*_*i*+1_ = *α* · *T*_*i*_, where *T*_*i*_ is the temperature used to compute the acceptance probability at iteration *i*.

**Step 9 (convergence check)**

If *T*_*i*+1_ < *t*_*f*_, stop and output the optimal solution $\overset{-}{S}$ and the set $\overset{-}{A}$. Otherwise reset *i* = 1, and return to Step 4.

In the above algorithm, the global optimal solution $\overset{-}{S}$ is saved in Step 5, which is the best solution found so far. And only if the neighborhood solution **S**′ is better than the optimal solution $\overset{-}{S}$, do we replace the optimal solution with the neighborhood solution.

It should be noted that if and only if $\overset{-}{A}=\phi$ after the algorithm terminates, the model has the optimal solution. Otherwise it can be deemed that the initial problem has no feasible solution, so then we should relax the link load constraints and/or land-use scale constraints appropriately and recalculate to obtain the optimal solution.

The method of solution for the lower-level traffic equilibrium model can be divided into two types, which are link-based and path-based. Compared to the link-based algorithm, the path-based algorithm can provide more traffic flow information with a higher computational efficiency [18, 19]. So we adopt the path-based gradient projection (pGP) algorithm to solve the low-level model. The following is a step-by-step description of the procedure for the pGP algorithm [18].

**Step 1 (initialization)**

Generate an initial path for each OD pair.

*Step  1.1*. Set *x*_*a*_(0) = 0, *t*_*a*_ = *t*_*a*_[*x*_*a*_(0)], for  all  *a* and *K*_*rs*_(0) = *ϕ*.

*Step  1.2*. Set iteration counter *n* = 1.

*Step  1.3*. Solve the shortest problem and get ${\overset{-}{k}}_{rs}\left(n\right)$. Update the path set:

(9) $$\begin{array}{c} K_{rs}\left(n\right)={\overset{-}{k}}_{rs}\left(n\right)\cup K_{rs}\left(n-1\right),\;\forall r,s. \end{array}$$

*Step  1.4*. Perform “All or No” assignment: $f_{{\overset{-}{k}}_{rs}}^{rs}=q_{rs}$, for  all  *r*, *s*.

*Step  1.5*. Calculate the flows on roads according to the flow on the path:

(10) $$\begin{array}{c} x_{a}\left(n\right)=\sum_{r\in R}\sum_{s\in S}\sum_{k\in K_{rs}(n)}f_{k}^{rs}\left(n\right)\delta_{ka}^{rs},\;\forall a. \end{array}$$

**Step 2 (column generation)**

Generate the shortest path based on the current link travel times and augment the set of generated path if it is new.

*Step  2.1*. Increment iteration counter *n* = *n* + 1.

*Step  2.2*. Update link travel times: *t*_*a*_(*n*) = *t*_*a*_(*x*_*a*_(*n* − 1)), for  all  *a*.

*Step  2.3*. Solve the shortest problem: ${\overset{-}{k}}_{rs}\left(n\right)$, for  all  *r*, *s*.

*Step  2.4*. Augment path ${\overset{-}{k}}_{rs}\left(n\right)$ into path set *K*_*rs*_(*n* − 1) if it is not already existed. If ${\overset{-}{k}}_{rs}\left(n\right)\notin K_{rs}\left(n-1\right)$, then $K_{rs}\left(n\right)={\overset{-}{k}}_{rs}\left(n\right)\cup K_{rs}\left(n-1\right)$, for all *r*, *s*. Otherwise, if ${\overset{-}{k}}_{rs}\left(n\right)\in K_{rs}\left(n-1\right)$, then tag the shortest path in the path set *K*_*rs*_(*n* − 1) as ${\overset{-}{k}}_{rs}\left(n\right)$ and set *K*_*rs*_(*n*) = *K*_*rs*_(*n* − 1).

**Step 3 (equilibration)**

Solve the path-formulated traffic assignment problem over the restricted set of paths generated thus far.

*Step  3.1*. Calculate first derivative path costs *d*_*k*_^*rs*^(*n*), $d_{{\overset{-}{k}}_{rs}}^{rs}\left(n\right)$ and second derivative path costs *s*_*k*_^*rs*^(*n*), where

(11) skrs(n)=∑a∈Ata′(n)(δkars−δk−rs(n)ars)2,  ∀k∈Krs(n),  ∀k≠k−rs(n),  ∀r,s,dkrs(n)=∑a∈Ata(n)δkars, ∀r,s,dk−rsrs(n)=∑a∈Ata(n)δk−rs(n)ars, ∀k∈Krs(n),          ∀k≠k−rs(n),  ∀r,s.

*Step  3.2*. Update the flows on the nonshortest paths:

(12) fkrs(n+1) =max⁡{[fkrs(n)−α(n)(skrs(n))−1(dkrs(n)−dk−rs(n)rs(n))],0},                 ∀k∈Krs(n),∀k≠k−rs(n),∀r,s,

where *α*(*n*) is the stepsize and it could be set as *α*(*n*) = function(*n*) = (*n*+1)^−1^.

*Step  3.3*. If *f*_*k*_^*rs*^(*n* + 1) = 0, then drop the path *k*: *K*_*rs*_(*n*) = *K*_*rs*_(*n*)∖*k*.

*Step  3.4*. Update the flows on the shortest paths:

(13) $$\begin{array}{c} f_{{\overset{-}{k}}_{rs}\left(n\right)}^{rs}\left(n+1\right)=q_{rs}-\sum_{k\in K_{rs},\;k\neq {\overset{-}{k}}_{rs}(n)}f_{k}^{rs}\left(n+1\right),\;\forall r,s. \end{array}$$

*Step  3.5*. Update link flows:

(14) $$\begin{array}{c} x_{a}\left(n+1\right)=\sum_{r\in R}\sum_{s\in S}\sum_{k\in K_{rs}(n)}f_{k}^{rs}\left(n+1\right)\delta_{ka}^{rs},\;\forall a. \end{array}$$

**Step 4 (termination)**

Terminate the algorithm if it satisfies the stopping criterion.

*Step  4.1*. If $\max_{r,s}\sum_{k\in K_{rs},\;k\neq {\overset{-}{k}}_{rs}(n)}f_{k}^{rs}\left(n\right)\left(d_{k}^{rs}\left(n\right)-d_{{\overset{-}{k}}_{rs}}^{rs}\left(n\right)\right){\left(q_{rs}d_{k}^{rs}\left(n\right)\right)}^{-1}\leq \varepsilon$, then terminate, output the link flows; otherwise, proceed to Step 2.

## 5. Numerical Examples

**Figure 3:**
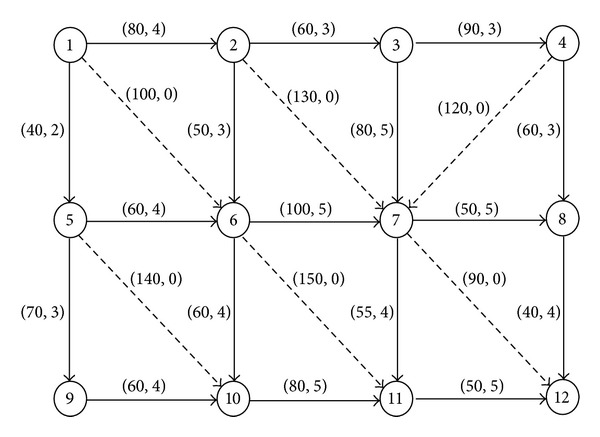
12-node transportation network.

In this section a transportation network, illustrated in Figure 3, is used to show the numerical results of the proposed model and solution method, which consists of 12 nodes and 5 OD pairs: *q*_1,11_ = 4900, *q*_1,12_ = 10000, *q*_2,8_ = 8000, *q*_2,12_ = 9000, and *q*_5,12_ = 8000. The 17 solid lines represent the existing links and the 6 dashed lines represent the candidate links. The length and initial grade of each link are given as the first and second values in parentheses, respectively. The available link grade s and its related information are shown in Table 1.

As mentioned previously, the construction cost of the link is determined by its initial and final link grade. We could regard the link construction cost function *I*_*a*_(*u*_*a*_, *u*_*a*_^0^) as

(15) $$\begin{array}{c} I_{a}\left(u_{a},u_{a}^{0}\right)=I_{a}\left(u_{a}\right)-I_{a}\left(u_{a}^{0}\right). \end{array}$$

From Table 1 we could conclude that if the link grade *u*_*a*_ is determined, the free-flow speed on the link, as the designed speed *v*_*a*_, can also be found (kilometers/hour), and thus the free-flow time on link *a* is

(16) $$\begin{array}{c} t_{a}^{0}=\frac{l_{a}}{v_{a}}. \end{array}$$

For the travel time function, *t*_*a*_ = *t*_*a*_^0^ · (1 + *α*(*x*_*a*_/*c*_*a*_)^*β*^), the parameters are uniformly set as *α* = 0.15, *β* = 4, thus the travel time on link *a* is

(17) $$\begin{array}{c} t_{a}=t_{a}^{0}\cdot {\left(1+\alpha \left(\frac{x_{a}}{c_{a}}\right)\right)}^{\beta }=\frac{l_{a}}{v_{a}}\cdot {\left(1+0.15\left(\frac{x_{a}}{c_{a}}\right)\right)}^{4}. \end{array}$$

The land-use amount function can be set specifically as

(18) $$\begin{array}{c} e\left(u_{a},u_{a}^{0}\right)=8\left(u_{a}-u_{a}^{0}\right)+30\left(L\left(u_{a}\right)-L\left(u_{a}^{0}\right)\right), \end{array}$$

where *L*(*u*_*a*_) refers to the number of lanes owned by link *a* at the link grade of *u*_*a*_. 

For the link load constraint, set *R*_max⁡_ = 0.85 for all links. The upper limit value of land amount *E* = 1000, the unit matching coefficient is *θ* = 100.

The heuristic parameters are fixed as follows: cooling rate *α* = 0.7, and the maximum number of iterations at each temperature value *N* = 10|*R*||*S*|. 

For these mentioned models and solution algorithms, the results can be achieved with the help of Visual C# 2005 programming. A personal computer with Intel DUO Core 2.0 Ghz CPU, 4 G RAM and Windows XP Professional operating system was used for the test. The optimal solution for the problem can be obtained within about 4 seconds. 

The travel times for each OD pair in the initial network and optimized network are listed in Table 2. Compared with the initial network, the OD travel times in the optimized network fell by 44.24–80.61%. The reconstruction of the transportation network significantly improves its performance. 

**Figure 4:**
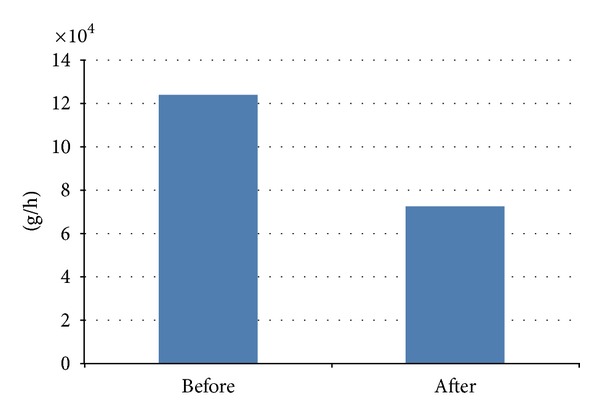
CO emission amount in network before and after optimization.

Figure 4 shows the information on the grades and loads of various links under the flow equilibrium state before and after optimization, where the information marked above the line is the initial link grade and load, and the information below the line is the corresponding information after optimization. From the figure we can determine that most link loads before optimization exceed the allowable range. The congestion is obvious in the links, especially in links such as (1,5), (2,3), (2,6), (5,9), and (6,10). These links have less flexibility and low elasticity for demand, which is not conducive to sustainable development of the transportation network. However, in the optimized network links (1,5), (2,3), (2,6), (5,6), (6,7), and (6,10) are upgraded, whilst at the same time constructing new links (1,6), (2,7), and (7,12). The loads of links with flows are in the range (0.29, 0.84), which all meet the related constraints. Thus, the new links play a good role in diverting the flows on the congestion links in the network and effectively improve the gridlock of the transportation network.

The total investment of the network improvement project is 192.8 million Yuan, and the land-use amount is 862. The total CO emission in the transportation network drops by 41.56%, reduced from 123,729.00 g/hour before optimization to 72,301.74 g/hour after optimization. It shows a significant improvement in environmental protection in the transportation network.

**Figure 5:**
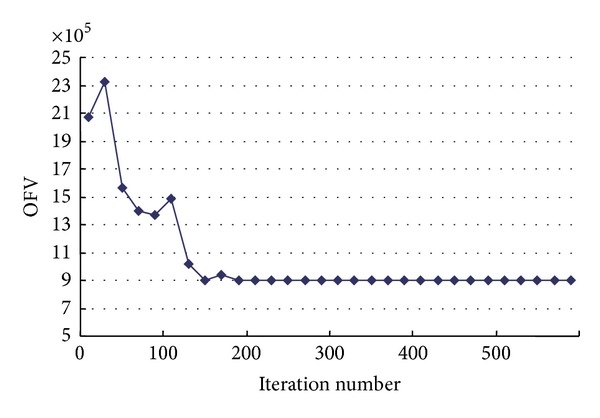
Objective value of upper model versus iteration number of SA.

**Figure 6:**
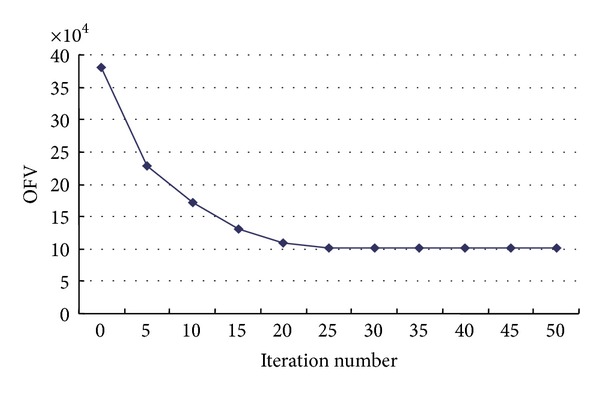
Objective value of lower model versus iteration number of GP.

In addition it can be seen from Figure 5 (OFV is the objective function value of the upper-level model) that that the SA-pGP algorithm determines the optimal solution after about 200 iterations. The algorithm convergence speed and convergence effect are really good, which also indicates that the SA algorithm proposed in this paper is effective.  Figure 6 shows that the objective function of the lower model decreases quickly with the number of iterations in the computation process of the GP algorithm (OFV is the objective function value of the lower-level model) and converges to an optimal solution by the 25–30th iteration.

## 6. Conclusions

This paper studied the sustainable MTNDP. Link-grade decision variables were used to discretize the mixed problem, based on which the bi-level programming model is developed. The upper model is built to minimize the CO emissions of vehicles on the links and also the total investment, in terms of land-use scale and link load constraints, the lower one is the deterministic traffic assignment model. A heuristic algorithm is proposed to solve the model, in which the SA algorithm and the path-based GP algorithm are used to solve the upper and lower models, respectively.

The numerical examples demonstrated that the optimization model and the solution method are effective, in which the SA is not only more efficient and robust with regard to the calculations, but it also rapidly converges to the optimal solution. The path-based GP algorithm calculates quickly and converges steadily. Compared to the initial transportation network, the network optimization significantly improved congestion and pollution in the network. Thereby, the bi-level programming model of the sustainable mixed transportation network design and its solution algorithm can be used to solve real transportation network design problems, and it could provide some scientific evidence for decision making during transportation network planning when considering sustainable development.

## Figures and Tables

**Table 1:** Decision variables of link grade and its corresponding information in China.

Link status variable *u*	0	1	2	3	4	5	6	7	8	9
Link grade	No-way	4th-level	3rd-level	2nd-level	2nd-level	1st-level	1st-level	Highway	Highway	Highway
Lane	0	2	2	2	4	4	6	4	6	8
Design speed	0	40	60	80	100	100	100	120	120	120
Capacity	0	1500	3000	4000	6000	15000	20000	30000	45000	60000
Construction cost (tens of thousands of Yuan/km)	0	80	200	500	800	1500	3000	4000	6000	7000

**Table 2:** Comparison of OD traveling times.

OD	Travel time in initial network (hour)	Travel time in optimized network (hour)	Gap (%)
(1, 11)	11.690	2.513	78.50
(1, 12)	12.664	3.018	76.17
(2, 8)	9.973	1.934	80.61
(2, 12)	11.720	2.354	79.92
(5, 12)	4.677	2.608	44.24
